# Effect of Geopolymerization Reaction on the Flexural Strength of Kaolin-Based Systems

**DOI:** 10.3390/ma17102223

**Published:** 2024-05-08

**Authors:** Binghuan Gao, Yangyang Li, Seongwan Jang, Hyeonjin Son, Heesoo Lee, Chang-Jun Bae

**Affiliations:** 1 Department of 3D Printing Materials, Korea Institute of Materials Science (KIMS), Changwon 51508, Republic of Korea; gaobinghuan@kims.re.kr (B.G.); liyangyang@kims.re.kr (Y.L.); relicum@kims.re.kr (S.J.); sonil21@kims.re.kr (H.S.); 2 Department of Materials Science and Engineering, Pusan National University, Busan 46241, Republic of Korea; 3 Department of Advanced Materials Engineering, University of Science & Technology (UST), Daejeon 34113, Republic of Korea

**Keywords:** geopolymerization reaction, reaction degree, N-A-S-H links, flexural strength

## Abstract

Geopolymers exhibit broad application prospects, including construction and radiation shielding, which require excellent mechanical performances. However, investigations on the nature of geopolymerization reactions and their consequential impact on mechanical performance are still vague. In this study, the effect of the major factors of Si/Al ratio and curing time on the geopolymerization reaction and flexural strength were studied based on the microstructure evolution and chemical bonding formation analyzed using the SEM, FTIR, peak deconvolution, and XRD methods. The microstructure of geopolymers was transferred from initially layered smooth particles of kaolinite to a 3D network porous structure, corresponding to sodalite. A spectrum exclusive to the geopolymer structure occurred at 973 cm^−1^, corresponding to the sodium aluminum silicate hydrate (N-A-S-H) links, the integral area of which represents the degree of geopolymerization reaction. Furthermore, a controllable reaction degree was achieved by adjusting the Si/Al ratio and curing time, where the maximum reaction degree of 55% was achieved at a Si/Al ratio of 1.94 when cured for 7 d. The correlation between the flexural strength and reaction degree was found to follow a proportional relationship, achieving a flexural strength of 21.11 MPa with a degree of 45%. This study provides insight into the development of mechanical strength through controlling the reaction process.

## 1. Introduction

In recent years, environmental issues have gradually attracted people’s attention, especially the climate anomalies caused by the greenhouse effect that have intensified year by year, making the requirement to reduce carbon emissions increasingly urgent. Among the various carbon emissions, the preparation and use of ordinary Portland cement is estimated to contribute to up to 8% [1]. Compared to traditional cement, geopolymer, a newly developed inorganic cementitious material, has been identified as an ideal alternative to ordinary Portland cement as it reduces CO_2_ emissions by around 60–80% [2]. Geopolymer cements have been developed using various recycled materials, such as fly ash [3], slag [4], and steel-making dust [5], which demonstrate comparable performances to ordinary Portland cement in terms of durability, mechanical properties, corrosion resistance, thermal stability, and energy efficiency [6].

Owing to its eco-friendly nature, recycling of industrial wastes, and excellent comprehensive performance, geopolymer has been developed at a booming speed. The synthesis of geopolymer involves dissolution, monomer reconstruction, and condensation initiated by an activator [7], which is called a geopolymerization reaction (GR). Researchers have found that the GR process varies significantly in various conditions, leading to a wide variety of properties of as-prepared geopolymers. For example, the geopolymerization reaction depends on many different factors, including the raw materials and reaction parameters, such as the molar ratio of Si/Al and Na/Al, curing temperature, and time, which follow an order of statistical importance with an increasing effect on GR: curing time, curing temperature, Na/Al molar ratio, and Si/Al molar ratio [8].

However, our understanding of the mechanism of the geopolymerization reaction is inconsistent or even controversial due to the variety of raw material and reaction conditions, as well as the uncertainty of the interacting components. It has been reported that the concentration of NaOH has a significant influence on the mechanical properties, where an optimum NaOH concentration (12 M) leads to a higher flexural strength [9]. Other researchers have found that there is a limit to the concentration of alkaline activator needed to achieve a high geopolymer content, because the excessive amounts of NaOH decrease the Si/Na ratio and retarded the polycondensation process [10]. Dinh et al. found that the most effective molar ratios (Si/Al = 3.5–4) demonstrated the highest compressive strength in the fly ash/ground granulated blast-furnace slag system [11]. Hence, it can be seen that the optimal performance of geopolymer is highly reliant on the material system due to the different reaction process.

Various raw materials have been used to develop geopolymers with excellent performances, among which kaolin and zeolite are commonly used. Zeolite is usually used for the preparation of functional geopolymers for specific applications due to its special microporous and mesoporous structures, such as the ion exchange, catalytic activity, and molecular sieve [12]. Compared to zeolite, kaolin is a relatively chemically stable mineral with good heat resistance and acid and alkali resistance, which are crucial and beneficial for the fundamental study of the geopolymerization reaction. In addition, kaolin has easy availability and cost advantages in geopolymer preparation due to its abundant resources. Therefore, as a simplified case study to investigate the reaction kinetics of geopolymer, using a single aluminosilicate raw material of kaolin is more favorable and efficient for clarifying the main parameters affecting and facilitating the reaction process. Various studies regarding kaolin-based geopolymers have been performed. The effect of the solid-to-liquid (S/L) ratio on kaolin-based geopolymer was reported, and it was found that the highest compressive strength was obtained when S/L was equal to 1 [13]. Hounsi et al. explored how the mechanical activation affected the curing process. Mechanically activated reactants were obtained by dry ball-milling of raw kaolin to achieve high compressive strength [14]. Although a large number of studies have been carried out on the kaolin system, in-depth understanding focusing on the mechanism is still lacking due to the performance-led research trajectory.

Furthermore, in order to deeply understand the reaction mechanism, a proper characterization technique is required to analyze the structural evolution and bonding formation. To evaluate the bonding state within geopolymers, the most commonly used techniques are nuclear magnetic resonance spectroscopy (NMR) and Fourier transform infrared spectroscopy (FTIR). Researchers have reported various studies based on the NMR test. Li et al. [15] investigated the reaction mechanism using longitudinal single-sided NMR. It was found that the Si/Al ratio affected the gelation and polymerization stages as well as the gel phase. Singh [16] studied the structural evolution using ^29^Si and ^27^Al MAS NMR. Greiser et al. [17] reported the fraction of geopolymeric gel in the reaction products using solid-state NMR. Despite the atomic-level evaluation of geopolymer bonding based on NMR method, the obtained signal could be inaccurate due to the nature of geopolymer materials. The alkali metal ions (e.g., sodium, potassium) can interact strongly with water molecules and affect NMR measurements. Geopolymer systems can exhibit complex NMR spectra with overlapping peaks due to multiple chemical environments and interactions.

In addition, FTIR is more widespread and more easily available compared to the NMR method. Therefore, in this study, FTIR was employed for the analysis of the geopolymerization reaction because it could identify functional groups and chemical bonds present in geopolymers, providing information about the bonding characteristics and chemical composition. Zhang et al. [18] applied Fourier transform infrared (FTIR) deconvolution analysis to quantitatively present the kinetic and structural evolution of geopolymers; their results indicated the formation of geopolymer products, as confirmed by a band shift from ~1080 to ~1000 cm^−1^. Rovnaník [19] observed the formation of geopolymer cementitious compounds, which confirmed the characteristics of Si-O-T (T is Si or Al) vibration at the FTIR absorbance peak within the 1300–900 cm^−1^ region. Nevertheless, given the differences in microstructure, thermally activated metakaolin has an amorphous phase and demonstrates greater reactivity compared to kaolin, which leads to a different GR process.

Given the numerous studies regarding geopolymers, most of them are application-oriented, focusing on the optimization of a specific property based on trial and error method. In-depth understanding of the geopolymerization reaction, including the reaction degree, kinetics, and the correlation between the degree of geopolymerization reaction and mechanical performance, still needs to be achieved. In this work, we are committed to the fundamental understanding of the reaction mechanism and reaction kinetics, and we explore the relationship between microscopic reactions and macroscopic properties. The effects of chemical and physical factors such as composition and curing time on the geopolymerization reaction were systematically investigated. The controllable reaction degree of GR was studied by characterizing the typical FTIR absorption peaks of the geopolymer structure. Then, the correlation between flexural strength and the reaction degree of geopolymerization was revealed. Since the geopolymers show great potential for various applications, especially the high-mechanical-performance field, this study provides a deep understanding of how to control the degree of the geopolymerization reaction to achieve the anticipated mechanical strength, which is beneficial for reducing the cost and improve the utilization efficiency of the raw materials. Based on this fundamental understanding, the next investigations of practical application of geopolymers will be carried out more smoothly to achieve excellent properties.

## 2. Materials and Methods

### 2.1. Materials

Kaolin powders (supplied by Sigma-Aldrich, Seoul, Republic of Korea) were the main aluminosilicate source used to prepare the geopolymers. Kaolin is primarily composed of kaolinite and trace amounts of minerals and oxides. Detailed information regarding their compositions is provided in Table 1. The weight percentages of silica and alumina in the kaolin powder were 59.74 wt.% and 36.28 wt.%, respectively, which resulted in an initial Si/Al ratio of 1.40. Silica powders (Sigma-Aldrich, Seoul, Republic of Korea, 99%) were used as an additional Si source to adjust the Si/Al molar ratio in the system. The detailed calculation method to adjust the Si/Al ratio is shown in Table S1. Table 2 shows the particle sizes of the two powders measured by a laser diffraction particle size analyzer (Coulter LS 13320, Beckman Coulter Inc., Miami, FL, USA), including the mean particle size (dm), d_10_, d_50_, and d_90_. NaOH (SAMCHUN, Seoul, Republic of Korea, 98%) beads were dissolved in distilled water to prepare an alkaline activator at a concentration of 6 mol/L.

### 2.2. Sample Preparation

The geopolymer specimens were prepared as following process: First, raw kaolin and silica powders were mixed homogeneously by mechanical stirring to form mixtures with the desired Si/Al molar ratios. Geopolymer pastes were obtained by adding NaOH solution (6 M) to the powder mixtures (solid/liquid = 1 g/1 mL) in the standard container of Thinky mixer (ARE-310, Tokyo, Japan) with a zirconia ball, followed by mixing at 2000 rpm for 2 min (30 s × 4). Subsequently, the paste was transferred to a syringe and centrifuged for 2 min at 2000 rpm to eliminate the trapped air. Finally, a pressure-driven robotic deposition apparatus (SHOTmini 200Sx, Musashi Engineering, Tokyo, Japan) was used to fabricate the specimens via the DIW process. To achieve high-quality printing, the key printing parameters, including the extrusion pressure, printing speed, nozzle diameter, and layer thickness, were specified as shown in Table S2. Specimens (pellet, diameter: 25 mm; thickness: 2 mm) were prepared to further investigate the geopolymerization under various conditions, as shown in Table 3.

### 2.3. Characterization

Scanning electron microscopy (JSM-6610, JEOL, Tokyo, Japan) was used to investigate the evolution of microstructure and morphology of the geopolymer specimens. Prior to observation, a gold layer was sputtered onto the sample surface for 1 min to improve the conductivity.

Flexural strength was measured with the ASTM F394-78 method [20], i.e., piston-on-three-ball test using a universal testing machine (Model RB301 UNITECH M, R&B Inc., Incheon, Republic of Korea) with a load cell (UM-K50, DACELL, Cheongju-si, Republic of Korea) under an operation speed of 0.5 mm/min. For each condition, three to five samples (pellet shape, diameter: 25 mm; thickness: 2 mm) were required to ensure the reliability, and the error bars represent the standard deviation.

Compressive strength was measured based on the ISO 604 method [21] using a universal testing machine (Instron-5982, Chicago, IL, USA) with a constant loading rate of 0.5 mm/min. For each condition, three samples (length: 10 mm; width: 10 mm; thickness: 4 mm) were required to ensure the reliability.

X-ray diffraction (XRD; D/Max 2500, RIGAKU, Auburn Hills, MI, USA) was performed to elucidate the phases of the synthesized geopolymers in the 2θ range of 10–80° at a scan speed and step size of 10 °/min and 0.02°, respectively; a Cu Kα radiation (wavelength: 0.15406 nm) source and glass holder were used.

Fourier transform infrared (FTIR) spectroscopy (Nicolet iS10, Thermo Scientific, Waltham, MA, USA) was performed to obtain the transmittance and absorbance spectra over the range of 4000–400 cm^−1^, with scanning 64 times with a resolution of 2 cm^−1^. For the investigation of GR, each sample was ground into powder.

**Figure 1:**
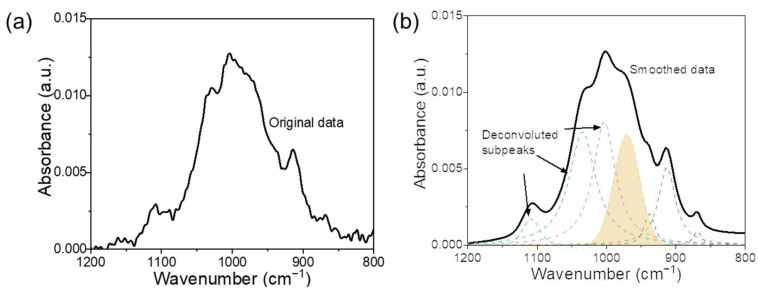
FTIR spectra of geopolymer. (**a**) Absorbance. (**b**) Schematic diagram of deconvolution of FTIR spectra.

The spectra obtained by FTIR spectroscopy were converted into a relative absorbance curve from the original transmittance value, followed by deconvolution within the range of 1200–800 cm^−1^ using the Peakfit (Version 4.12) software, where the Gaussian mode, variable shape, and widths were applied. Fitting was performed based on previously reported procedures [18,22] where a built-in self-fitting function was used to determine the number and position of sub-peaks. The primary principle of the fitting process is to reduce the number of subpeaks as well as to maintain a regression coefficient R^2^ exceeding 0.999. Figure 1a,b show schematic illustrations of the deconvolution analysis.

## 3. Results and Discussion

### 3.1. Microstructure and FTIR Spectra of Geopolymers

**Figure 2:**
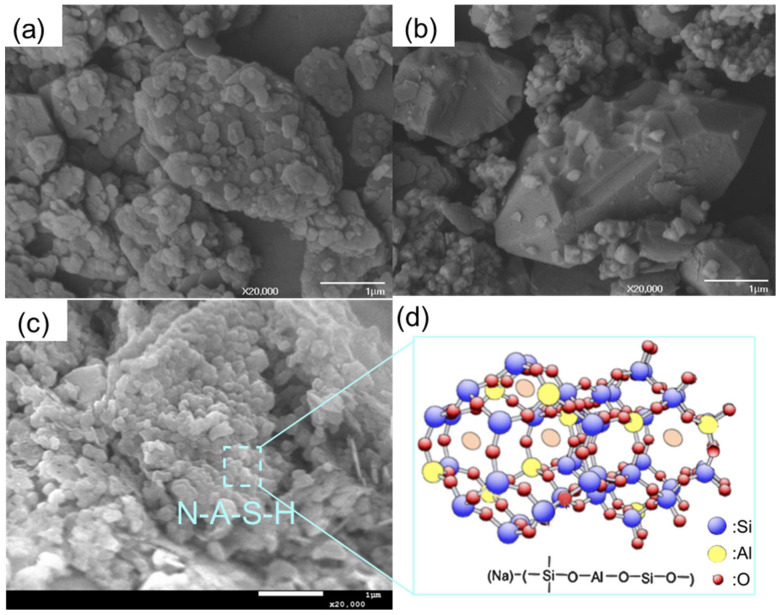
Microstructure and FTIR spectra of raw materials and geopolymers. (**a**) Kaolin powders. (**b**) Silica powders. (**c**) Morphology of N-A-S-H gel of the geopolymer. (**d**) The modified atomic 3D network of N-A-S-H gel, where the pink oval represents the Na+ for charge balance [27].

The microstructures of unreacted kaolin and silica powders are shown in Figure 2a and Figure 2b, respectively. Kaolin particles have a layered structure with a smooth surface, whereas silica particles have an irregular shape that differs from the shape of spherical sodalite particles [23]. It has been reported that the geopolymerization reaction involves dissolution, monomer reconstruction, and condensation initiated by an activator [7], correspondingly, the microstructure also changed as a result of the structural reorganization. First, the surfaces of kaolinite particles were attacked by hydroxyl groups (OH^−^), resulting in their layer-by-layer dissolution into small aluminum and silicon species [24]. Furthermore, the dissolved species aggregated and formed particles which contained the 3D network of N-A-S-H links shown in Figure 2c,d. Moreover, pores were inevitably formed during this process.

Figure 2c shows the uniform N-A-S-H bonding of the geopolymeric gel, which resulted in tightly packed particles (rectangular region) on the smooth surface of kaolinite. As reported previously [25], aluminosilicates reacted with Na-based alkaline solution and yielded the main product of N-A-S-H gel, which was formulated as (M_n_-(SiO_2_)-(AlO_2_)_n_.wH_2_O). As the main intermediate products of the geopolymerization reaction, the SiO_4_ and AlO_4_ tetrahedra were produced after the dissolution process, which coordinated with each other through oxygen bonding to form the Si-O-T (T = Si or Al) links. The formation of Si-O-T (T = Si or Al) links followed a specific stoichiometry, which was determined by the overall chemical environment. Moreover, the negative charge was balanced by positive alkaline cations (Na^+^ and K^+^) [26]. The corresponding 3D atomic structure of the N-A-S-H bonding [27] is represented in Figure 2d, where Si is dominated by Q4(3Al) and Q4(2Al) units.

**Figure 3:**
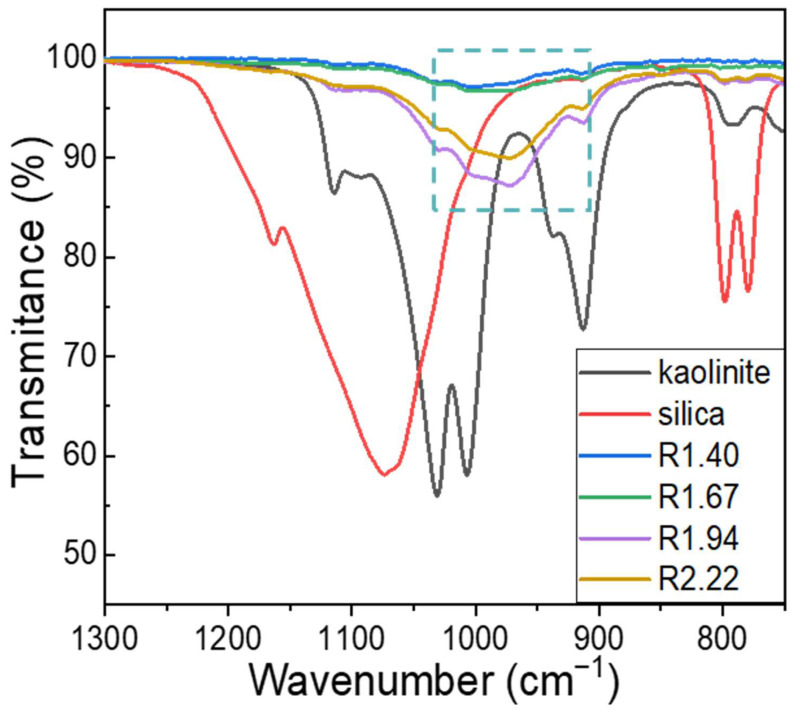
FTIR spectra of raw powders and geopolymers with various Si/Al molar ratios. The characteristic peaks of raw kaolinite (1031 cm^−1^) and silica (1073 cm^−1^) are replaced by those of geopolymers (rectangle region, 973 cm^−1^) for different Si/Al molar ratios.

As one of the most effective and readily available methods for quantifying GRs, FTIR is widely used to identify gel bonds and the environment of the predominant Si and Al elements in geopolymeric structures [28]. Figure 3 shows the characteristic peaks of silica, kaolinite, and the geopolymers, where maximum transmittances were indicated at 1073, 1031, and 973 cm^−1^, respectively, thus confirming the structural change and revealing the dependence of the GR on the Si/Al molar ratio. The characteristic banding of raw kaolinite and silica disappeared after the GR and was replaced by a new banding exclusive to the geopolymers [29]. In the rectangular region, the peak located around 980 cm^−1^ indicated the asymmetric stretching vibration of Si-O-T links in the N-A-S-H gel [30]. The intensity of the peak depended on the Si/Al molar ratio owing to the different GR degrees, which were determined by the number of Si-O-T links. The formation of the network structure represented by the FTIR peaks would potentially impact the properties of the geopolymer, and will be described in the following section.

### 3.2. Effect of Si/Al Molar Ratio on Geopolymerization Reaction

**Figure 4:**
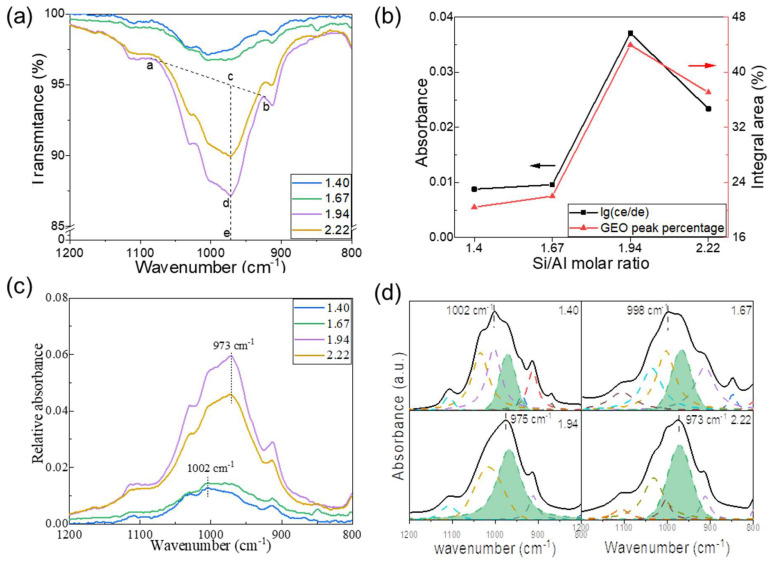
Effect of Si/Al molar ratio on the geopolymerization reaction. (**a**) Transmittance spectra of FTIR of geopolymers as well as the plot of Lambert–Beer law. (**b**) Comparison between Lambert-Beer law plot (black curve) and the percentage of integral area of geopolymer peaks by deconvolution. (**c**) The relative absorbance of FTIR spectra of geopolymers. (**d**) Deconvolution spectra of Si-O-T bands of geopolymers with different Si/Al molar ratios over 800–1200 cm^−1^.

Three-dimensionally printed geopolymers with different chemical compositions were used to analyze the degree of geopolymerization based on Equation (1), as shown by the intensities of the FTIR spectra. Figure 4a shows the transmittance of the FTIR spectra of the geopolymers cured for 24 h as a function of the Si/Al molar ratio. The principal new bonds of Si-O-T within 990–960 cm^−1^ were related to the asymmetric stretching vibration in the geopolymer network, which was sensitive to the Si/Al molar ratio [31]. The intensity of the transmittance depended on the amount of geopolymer products. For further quantification of the FTIR spectra with varying Si/Al molar ratios, the relative absorbance (black curve) based on the modified Lambert–Beer law is presented in Figure 4b.

(1) $$A=\mathrm{lg}\left(I_{0}/I\right)=\mathrm{lg}\left(ce/de\right)$$

where *a, b*, *c*, *d*, and *e* are the left shoulder, right shoulder, crossing point, maximum transmittance, and *x*-axis, respectively (Figure 4a). The relative absorbance reached a maximum at a ratio of 1.94, i.e., the maximum degree of GR under these conditions, which is consistent with previous results [32].

The relative absorbance of the new geopolymer bands within the range of 1200–800 cm^−1^ is shown in Figure 4c; it could be deconvoluted to determine the mechanism of the GR based on different compositions. The new peak shifted to a lower wavenumber from 1002 to 973 cm^−1^ as the Si/Al molar ratio increased because of the tetrahedralization of Al bonded to Si from the original kaolinite to the final geopolymer structure [18]. In addition, amorphous aluminosilicates can present a similar shift in the main T-O-T links due to the weakened Al-O bonds with respect to the Si-O [33] and T-O interatomic distances, where the mean T–O distance decreases as the Si/Al ratio increases because of the shorter Si-O (~1.61 Å) and longer Al-O (~1.8 Å) [34]. The deconvoluted sub-peaks shown in Figure 4d correspond to various structures and bonds. The curves filled with green color are attributed to the Si-O-T component at 973 cm^−1^ of the geopolymer products. The percentages of the integral areas of the geopolymer sub-peaks are summarized in Figure 4b (red curve), and are identical to the calculation results based on the Lambert–Beer law, thus verifying the complex role of the Si/Al ratio in the reaction. The addition of silica facilitates geopolymerization within a certain range of Si/Al; however, redundant silica serves as a defect in the mixture and impedes its progress [24].

### 3.3. Effect of Reaction Time on Geopolymerization Reaction

**Figure 5:**
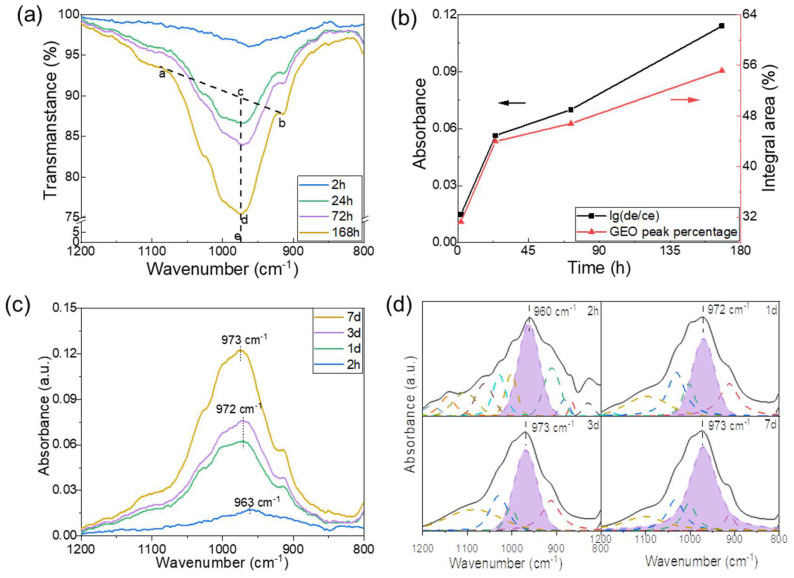
Effect of curing time on the geopolymerization reaction. (**a**) Transmittance spectra of FTIR of geopolymers as well as the plot of Lambert–Beer law. (**b**) Comparison between Lambert–Beer law plot (black curve) and the percentage of integral area of geopolymer peaks by deconvolution. (**c**) The absorbance of FTIR spectra of geopolymers. (**d**) Deconvolution spectra of Si-O-T bands of geopolymers with different curing times over 800–1200 cm^−1^.

Additionally, we further explored the progress of GR regarding the time dimension, as shown in Figure 5. The FTIR spectroscopy results show decreasing transmittance (Figure 5a) and increasing absorbance (Figure 5c) as the curing time increased, which was due to the enhanced GR, resulting in a more geopolymeric structure and typical characteristic bands at 973 cm^−1^. The main peak shifted to higher wavenumbers as the curing time increased because of the increased amount of dissolved Si. The initial dissolution of aluminosilicate began with a high level of Al, which generated a dealuminated layer on the particle surface, followed by the stoichiometric dissolution of Al and Si monomers from the particle [35]. Therefore, an Al-rich environment was formed in the initial stage because of the preferential solubility of Al, which resulted in a lower Si/Al molar ratio in the existing N-A-S-H networks and lower wavenumbers [31]. In the later stage, the environment for gel formation was abundant in Si since Si had dissolved, thus promoting a shift in the main band to higher wavenumbers, i.e., from 960 to 973 cm^−1^.

Similarly, Figure 5b (black curve) shows the correlation between the absorbance determined using Equation (1) and the curing time. An approximately linear positive correlation is presented, which is consistent with the results of a previous study, i.e., the transmittance of the Si-O-T peak decreased with age, which resulted in a greater intensity of the absorption peak [13]. The deconvolution of the geopolymers shown in Figure 5d exhibited a positive effect of the curing time on the GR, which was based on the enhanced integral area of the geopolymeric sub-peaks, as shown in the purple area. The percentage of the integral area summarized in Figure 5b increased with the curing time, thus revealing the continuous formation of new geopolymer products. Mo et al. [19,36] discovered that the longer curing times of geopolymer mixtures extended the reaction process and facilitated the formation of geopolymeric gels.

### 3.4. Mechanical Property of Kaolin-Based Geopolymers

Various investigations pertaining to improving the mechanical strength of geopolymers have been conducted, most of which have involved the effects of different experimental conditions [19,37] and compositions [8], whereas the intrinsic developing route of mechanical properties has not been revealed. In particular, the degree of the GR and its potential contribution to the excellent mechanical properties have yet to be elucidated. The evolution of the mechanical properties of geopolymers can be fundamentally understood by detecting the correlation between the GR and flexural strength. Moreover, a mathematical model was developed to quantify the mechanical evolution all over the process, where the high reliability of fitting was determined by the R^2^ value to achieve accurate functions related to the development of flexural strength.

**Figure 6:**
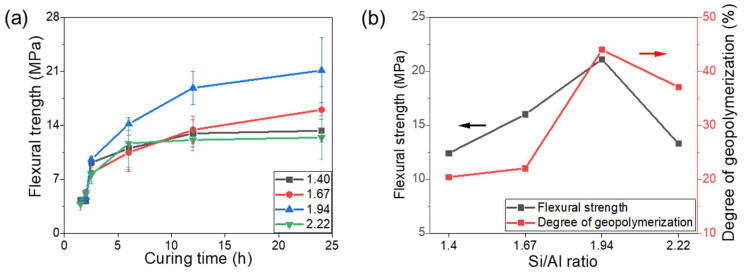
(**a**) Flexural strength of geopolymers with different Si/Al molar ratios as a function of curing time. (**b**) The correlation between the degree of geopolymerization and the flexural strength as functions of Si/Al ratio.

The flexural strengths of the various geopolymers exhibited logarithmic growth as a function of the curing time. In particular, it increased significantly within the first 24 h and then stabilized, as shown in Figure 6a. The geopolymer with a Si/Al molar ratio of 1.94 showed superior mechanical behavior of 21.11 MPa after 1 d curing owing to its higher degree of GR, which resulted in more N-A-S-H links and a stronger structure. Based on the deconvolution of the FTIR spectra, the correlation between the GR degree and the flexural strength of the geopolymers revealed an intrinsic positive dependence on the Si/Al ratio, as shown in Figure 6b. The highest GR degree resulted in the maximum flexural strength at a ratio of 1.94. Outliers were generated at a ratio of 2.22 due to the defects formed by excess silica powders, which disrupted the dense N-A-S-H structure and decreased the flexural strength [24].

## 4. Conclusions

Kaolin-based geopolymers were synthesized to investigate the geopolymerization reaction based on various conditions, including the Si/Al ratio and curing time, to fundamentally understand the nature of the GR process. The GR process was found to achieve the maximum reaction degree at a Si/Al ratio of 1.94 with an extended curing time. A longer curing time facilitated the formation of a larger amount of geopolymeric gels (N-A-S-H links). From 2 h to 168 h, the integral area of the geopolymeric gels increased from 31% to 55%. Furthermore, the correlation between the reaction degree and the flexural strength was revealed. A higher reaction degree led to the formation of a higher geopolymer content and a more crosslinked structure, thus producing a higher flexural strength. A flexural strength of 21.11 MPa was achieved with a reaction degree of 45% at the ratio of 1.94 when cured for 1 d. These findings provide an effective strategy for the applications of geopolymers, which could be developed for fields that require high mechanical strength, such as geopolymer-based concrete, geopolymer-based composites, and anti-erosion geopolymer coatings.

## Figures and Tables

**Table 1:** Chemical composition of kaolin (wt.%) based on XRF analysis.

Compound	Value	Unit
SiO_2_	59.7429	wt.%
Al_2_O_3_	36.2849	wt.%
TiO_2_	2.1513	wt.%
Fe_2_O_3_	1.1347	wt.%
K_2_O	0.2057	wt.%
P_2_O_5_	0.1356	wt.%
SO_3_	0.1055	wt.%
CaO	0.0741	wt.%
Na_2_O	0.0736	wt.%
Cr_2_O_3_	0.0291	wt.%
Ga_2_O_3_	0.0138	wt.%
ZnO	0.0123	wt.%
NiO	0.0115	wt.%
ZrO_2_	0.0098	wt.%
SrO	0.0093	wt.%
CuO	0.0059	wt.%

**Table 2:** Particle size of raw powders (µm).

Powder	d_m_	d_10_	d_50_	d_90_
Kaolin	0.902	0.213	0.494	2.075
SiO_2_	1.719	0.281	1.186	4.149

**Table 3:** Experimental conditions of geopolymerization.

Si/Al Ratio	Curing Time (h)	Curing Temperature (°C)
1.40	2, 24, 72, 168	80
1.67	2, 24, 72, 168	80
1.94	2, 24, 72, 168	80
2.22	2, 24, 72, 168	80

## Data Availability

Data are contained within the article and Supplementary Materials.

## Supplementary Materials

The following supporting information can be downloaded at: https://www.mdpi.com/article/10.3390/ma17102223/s1, Figure S1. XRD of geopolymers with different Si/Al ratios and curing times; Figure S2. XRD of geopolymers with different Si/Al ratios and curing times; Table S1. The calculation of specific Si/Al ratio by adding SiO_2_; Table S2. Printing parameters for sample reparation by DIW. References [38,39] are cited in the supplementary materials.
