# Supplementary material for: Effect of Geopolymerization Reaction on the Flexural Strength of Kaolin-Based Systems
Source: Materials (Basel). 2024 May 8;17(10):2223. doi: 10.3390/ma17102223 (PMC11122983; doi:10.3390/ma17102223)
Supplement: Supplementary file 1 [file materials-17-02223-s001.zip › materials-2974227-supplementary.pdf]

Supplement

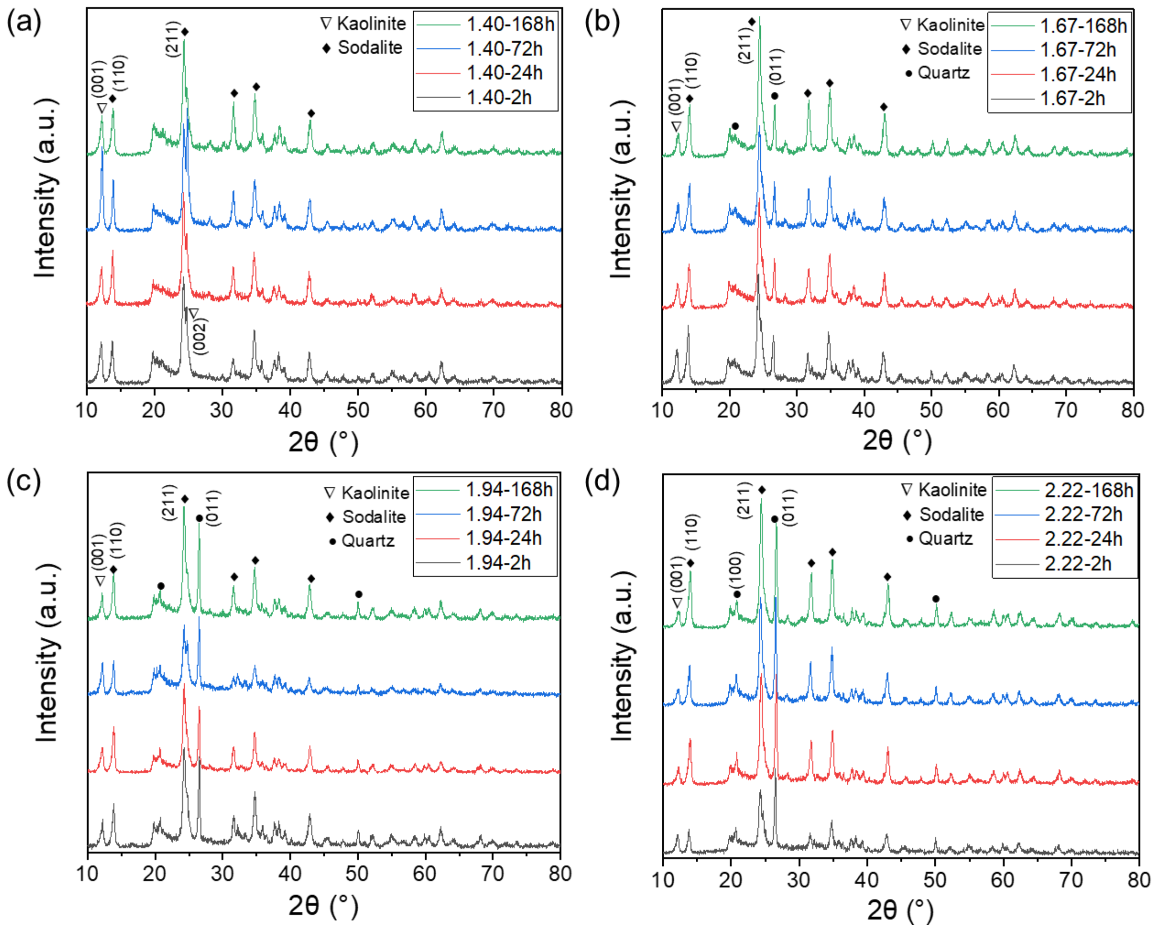

Figure S1. XRD of geopolymers with different Si/Al ratios and curing times.

To elucidate the phase transformation of kaolinite during geopolymerization, geopolymer samples with different Si/Al ratios and which were cured for different periods were investigated using XRD. Figure S1a, b, c, and d show the XRD patterns of geopolymers with Si/Al ratios of 1.40, 1.67, 1.94, and 2.22, respectively, cured for periods ranging from 2 to 168 h. XRD patterns of all the samples show characteristic peaks at 13.9°, 24.26°, and 35°, confirming the occurrence of the geopolymerization reaction. It has been reported that the peaks at 13.9°, 24.26°, and 35° are attributed to sodalite and only appear in geopolymer samples [1]. In addition, the intensity of the (011) peak at 26.64° of quartz increases with the increasing Si/Al ratio owing to the addition of silica. For all the different ratios, the (001) and (002) peaks attributed to kaolinite weaken and even disappear, while the (110) and (211) peaks attributed to sodalite appear and strengthen as geopolymerization

proceeds. In addition, the intensity of kaolinite (001) and (002) peaks, then decreases gradually with the increasing Si/Al ratio, implying that higher silica contents promote the geopolymerization reaction. Moreover, the intensity of the characteristic peaks attributed to kaolinite gradually decreases with increasing curing time from 2 h to 168 h, while the intensity of the sodalite characteristic peaks increases. This is in line with the results of FTIR analysis, involving a longer curing time and more geopolymerization reactions, producing more sodalite with a 3D silicaluminate network structure [2].

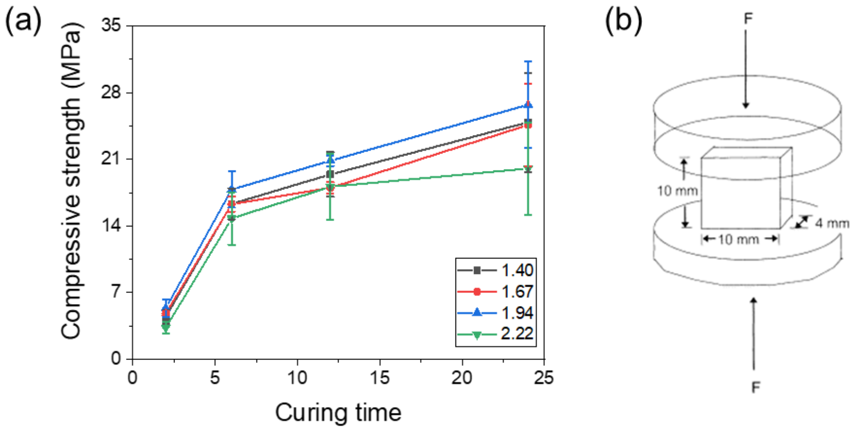

Figure S2. XRD of geopolymers with different Si/Al ratios and curing times.

The compressive strengths of geopolymers with different Si/Al ratios and curing times were measured according to ISO 604, as shown in Fig. S2. Fig. S2a illustrates the development of compressive strength, which is consistent with the flexural strength. The compressive strength increased as the curing time extended. The compressive strength increased as the curing time extended; it increased rapidly in the initial stage and then increased slowly until reaching a stable state. Fig. S2b shows the geometry of the tested specimens and the loading directions along the sample during the test.

Si/Al adjustment: According to the chemical composition of kaolin powder measured by the XRF method, it could be calculated that the original Si/Al ratio was 1.40. For example, if there is 1 mol of kaolin (M: 256.16) powder (256.16 g), the mass of SiO_2 is 154.2323 g, which is equal to 2.57 mol; the mass of Al_2O_3 is 93.6731 g, which is equal to 0.9184 mol; and the Al element is 1.8367 mol. Therefore, the Si/Al molar ratio is equal to 2.57/1.8367=1.40. The other ratios of the mixture were prepared by adding a certain amount of SiO_2 powder at an interval of 0.5 mol based on calculation shown in the following table.

Table S1. The calculation of specific Si/Al ratio by adding SiO_2.

Target Si/Al ratio	1mol kaolin (Si/Al)	Addition of SiO_2 (mol)
1.40	2.57/1.8367	x=0
1.67	(2.57+x)/1.837	x=0.5
1.94	(2.57+x)/1.837	x=1
2.22	(2.57+x)/1.837	x=1.5

After mixing homogeneously using the mechanical stirring method, the as-prepared mixtures were achieved and used for the experiment.

Table S2. Printing parameters for sample reparation by DIW.

Parameters	P (MPa)				V (mm/s)	D (mm)	t (mm)
	1.40	1.67	1.94	2.22			
Value	0.08	0.06	0.05	0.04	8	0.84	0.7

References

1. Shafiq, N.; Nuruddin, M.F.; Khan, S.U.; Ayub, T. Calcined kaolin as cement replacing material and its use in high strength concrete. Constr. Build. Mater. 2015, 81, 313-323.
2. Ma, W.; Yi, Y.; Fang, M.; Li, C.; Li, J.; Liu, W. Study on the synthesis mechanism of sodalite, gismondine, and zeolite-P1 zeolite materials from ladle furnace slag and fly ash. Sci. Rep. 2023, 13, 3232.
